# Steady-State Motion Visual Evoked Potential (SSMVEP) Enhancement Method Based on Time-Frequency Image Fusion

**DOI:** 10.1155/2019/9439407

**Published:** 2019-05-21

**Authors:** Wenqiang Yan, Guanghua Xu, Longting Chen, Xiaowei Zheng

**Affiliations:** ^1^School of Mechanical Engineering, Xi'an Jiaotong University, Xi'an, China; ^2^State Key Laboratory for Manufacturing Systems Engineering, Xi'an Jiaotong University, Xi'an, China

## Abstract

The steady-state motion visual evoked potential (SSMVEP) collected from the scalp suffers from strong noise and is contaminated by artifacts such as the electrooculogram (EOG) and the electromyogram (EMG). Spatial filtering methods can fuse the information of different brain regions, which is beneficial for the enhancement of the active components of the SSMVEP. Traditional spatial filtering methods fuse electroencephalogram (EEG) in the time domain. Based on the idea of image fusion, this study proposed an SSMVEP enhancement method based on time-frequency (T-F) image fusion. The purpose is to fuse SSMVEP in the T-F domain and improve the enhancement effect of the traditional spatial filtering method on SSMVEP active components. Firstly, two electrode signals were transformed from the time domain to the T-F domain via short-time Fourier transform (STFT). The transformed T-F signals can be regarded as T-F images. Then, two T-F images were decomposed via two-dimensional multiscale wavelet decomposition, and both the high-frequency coefficients and low-frequency coefficients of the wavelet were fused by specific fusion rules. The two images were fused into one image via two-dimensional wavelet reconstruction. The fused image was subjected to mean filtering, and finally, the fused time-domain signal was obtained by inverse STFT (ISTFT). The experimental results show that the proposed method has better enhancement effect on SSMVEP active components than the traditional spatial filtering methods. This study indicates that it is feasible to fuse SSMVEP in the T-F domain, which provides a new idea for SSMVEP analysis.

## 1. Introduction

To improve the comfort of light-flashing stimulation, we proposed a steady-state motion visual evoked potential (SSMVEP) method to replace light-flashing stimulation with motion stimulation in the previous study [1]. In this study, the SSMVEP signal was selected as a research object. The SSMVEP collected from the scalp will be contaminated by a variety of artifacts, such as the electrooculogram (EOG) and electromyogram (EMG). Consequently, the requirements for subsequent signal processing are high. In electroencephalogram (EEG) signal processing, using multichannel EEG is beneficial and effective [2, 3]. In the EEG literature, the method for linearly fusing the multilead signals into single-channel or multichannel signals is called spatial filtering. Spatial filtering combines the EEG information of different brain regions, which enhances the active components of the EEG [4, 5].

At present, the main methods used in EEG spatial filtering are average fusion, native fusion, bipolar fusion, Laplacian fusion, common average reference (CAR) fusion, canonical correlation analysis (CCA) fusion, minimum energy fusion, and maximum contrast fusion [4–6]. Average fusion is obtained by averaging all electrode signals, and a bipolar fusion signal is obtained by subtracting the two electrode signals [7, 8]. It is called native fusion if only one of the electrode signals is analyzed. Laplacian fusion is a centered electrode signal minus the sum of four electrodes at the same distance from their surroundings. CAR fusion is another commonly used EEG spatial filtering method that enhances the signal-to-noise ratio (SNR) of the selected electrode signal by subtracting the mean of all electrode signals from the selected electrode signal. Dennis et al. studied the enhancement effects of standard ear reference, CAR fusion, and Laplacian fusion on the active components of EEG. The results show that the Laplacian fusion achieved the best fusion effects on EEG active components [9]. Friman et al. proposed the minimum energy fusion and maximum contrast fusion methods [5]. The minimum energy fusion was achieved by minimizing the noise energy, and the maximum contrast fusion was achieved by maximizing the EEG SNR. In the study, Friman et al. compared the effects of average fusion, native fusion, bipolar fusion, Laplacian fusion, minimum energy fusion, and maximum contrast fusion on steady-state visual evoked potential (SSVEP) signals. Among these, the enhancement effect of average fusion was worst, and the enhancement effect of minimum energy fusion was best. CCA fusion is used to study the linear relationship between two groups of multidimensional variables and is also a commonly used spatial filtering method [6].

Since Friman et al. demonstrated that minimum energy fusion and maximum contrast fusion had better fusion effects and did not compare minimum energy fusion and maximum contrast fusion with CCA fusion, this study only compared the enhancement effects of minimum energy fusion, maximum contrast fusion, CCA fusion, and the proposed method on the active components of the SSMVEP. Minimum energy fusion, maximum contrast fusion, and CCA fusion are used to analyze multidimensional EEG signals in the time domain. This study aims to investigate whether electrode signals can be fused in the time-frequency (T-F) domain. The method of image fusion can fuse multiple images into one, thus improving the image quality [10–12]. This study transformed EEG from the time domain to the T-F domain and analyzed multidimensional EEG with the idea of image fusion, to improve the enhancement effect of existing spatial filtering methods on SSMVEP active components. Firstly, two electrode signals were transformed from the time domain to the T-F domain via short-time Fourier transform (STFT). Then, two T-F images were decomposed by two-dimensional multiscale wavelet decomposition, and both the high-frequency coefficients and low-frequency coefficients of the wavelet were fused by specific fusion rules. After two-dimensional wavelet reconstruction, two images could be fused into one image. The fused image was filtered via mean filter, and the fused time-domain signal could be obtained by inverse STFT (ISTFT). The experimental results show that the proposed method based on T-F image fusion can enhance the SSMVEP better than the traditional time-domain analysis method.

## 2. Methods

### 2.1. Subjects

Six males and four females (20–28 years old) were recruited as subjects for this study. The participants were healthy and had normal colour and visual perception.

### 2.2. Experimental Equipment

g.USBamp (g.tec, Austria) was used to collect the EEG. The sampling frequency of the equipment is 1200 Hz. A g.USBamp EEG amplifier and g.GAMMAbox active electrode system were combined to form the experimental platform. Prior to the experiment, the reference electrode was placed at the left ear of the subjects, and the ground electrode Fpz was placed at the forehead. EEGs were collected from the following six channels: PO7, Oz, PO8, PO3, POz, and PO4.

### 2.3. Experimental Step

A checkerboard of radial contraction-expansion motion was used as a stimulation paradigm, and the details of the paradigm can be found in Reference [1]. The stimulus paradigms were presented on a screen at a refresh rate of 144 Hz, and the subjects were positioned 0.6–0.8 m from the screen. The experiment included six blocks, each containing 20 trials corresponding to all 20 targets. The stimuli were arranged in a 5×4 matrix, and the horizontal and vertical intervals between two neighboring stimuli were 4 cm and 3 cm, respectively. The stimulus frequencies were 7–10.8 Hz with a frequency interval of 0.2 Hz, and the radius of the stimuli was 60 pixels. Each trial lasted 5 s, separated by an interval of 2 s. Between two blocks, the subjects were allowed to rest properly. Twenty targets were simultaneously presented on the screen and numbered 1 to 20. Before the stimulus began, one of the 20 serial numbers appeared below the corresponding target, indicating the focus target.

### 2.4. Preprocessing of EEG Data

The corresponding EEG data segments were extracted in accordance with the trial start and end times. The MATLAB library function *detrend* was used to remove the linear trends for each channel. Chebyshev bandpass filtering of 0.5–50 Hz was used to remove low-frequency drifts and high-frequency interferences.

### 2.5. Significance Test

The data were expressed as mean values. A paired-sample *t*-test was used to determine the significance. Statistical significance was defined as *p* < 0.05.

### 2.6. Spatial Filtering Methods: Minimum Energy Fusion, Maximum Contrast Fusion, and CCA Fusion

For EEG data *Y* recorded from multiple electrode leads, the spatial filtering method obtains new data by linearly summing each of the electrode signals with spatial filter coefficients *W*. A spatially filtered signal can be expressed as

(1) $$\begin{array}{c} Z=W^{T}Y. \end{array}$$

The spatial filter coefficients *W* for minimum energy fusion, maximum contrast fusion, and CCA fusion are described below.

#### 2.6.1. Minimum Energy Fusion

For a visual stimulus frequency *f*, the SSMVEP signal recorded by the *i*th electrode can be expressed as follows:

(2) $$\begin{array}{c} y_{i}=\overset{n}{\underset{j=1}{\sum }}a_{i,j}\sin\left(2j\pi ft+\phi_{i,j}\right)+e_{i}\left(t\right), \end{array}$$

where *n* represents the number of harmonics and *a*_*i*,*j*_ and *ϕ*_*i*,*j*_ represent the amplitude and phase of the *j*th harmonic component, respectively. The model decomposes the signal into the sum of the SSMVEP induced by the visual stimulus and the noise *e*_*i*_(*t*) generated by the electromyogram (EMG), electrooculogram (EOG), and other components. Thus, equation (2) can be expressed as

(3) $$\begin{array}{c} y_{i}=S_{i}+e_{i}, \end{array}$$

where

(4) $$\begin{array}{c} S_{i}=a_{i}X,\\ X=\left[X_{1},X_{2},\ldots ,X_{n}\right]. \end{array}$$

The submatrix *X*_*n*_ is composed of sin(2*nπft*) and cos(2*nπft*), and *a*_*i*_ represents the amplitude of SSMVEP at its stimulus frequency and harmonics. We assume that the signal matrix recorded by *N* electrodes is *Y* = [*Y*_1_ *Y*_2_ … *Y*_*N*_], where each column corresponds to an electrode channel. *Y* can be projected to the SSMVEP space through a projection matrix *Q*, namely,

(5) $$\begin{array}{c} S=QY. \end{array}$$

Reference [13] gives the solution of *Q* as

(6) $$\begin{array}{c} Q=X{\left(X^{T}X\right)}^{-1}X^{T}. \end{array}$$

Thus, the noise signal can be expressed as

(7) $$\begin{array}{c} Y^{\prime }=Y-QY. \end{array}$$

The minimum energy fusion obtains the spatial filter coefficients *W* by minimizing the noise energy, namely,

(8) $$\begin{array}{c} \underset{W}{\min}{\left\|Y^{\prime }W\right\|}^{2}=\underset{W}{\min}W^{T}Y^{\prime }Y^{\prime }W. \end{array}$$

The above minimization problem can be obtained by decomposing the eigenvalues of the positive definite matrix *Y* ^′*T*^ *Y*′. After decomposition, *N* eigenvalues and corresponding eigenvectors can be obtained. The spatial filter coefficient *W* is the eigenvector corresponding to the smallest eigenvalue (MATLAB code for minimum energy fusion and test data is provided in Supplementary Materials (available here)).

#### 2.6.2. Maximum Contrast Fusion

The goal of maximum contrast fusion is to maximize SSMVEP energy while minimizing noise energy. The SSMVEP energy can be approximated as *W* ^*T*^ *Y* ^*T*^ *YW*. Therefore, the maximum contrast fusion can be achieved by the following maximizing equation:

(9) $$\begin{array}{c} \underset{W}{\max}\frac{{\left\|YW\right\|}^{2}}{{\left\|Y^{\prime }W\right\|}^{2}}=\underset{W}{\max}\frac{W^{T}Y^{T}YW}{W^{T}Y^{\prime T}Y^{\prime }W}. \end{array}$$

Here, *Y* and *Y*′ are the same as in Section 2.6.1. Equation (9) can be solved by generalized eigenvalue decomposition of *Y* ^*T*^ *Y* and *Y* ^′*T*^ *Y*′. The spatial filter coefficient *W* is the eigenvector corresponding to the largest eigenvalue (MATLAB code for maximum contrast fusion and test data is provided in Supplementary Materials (available here)).

#### 2.6.3. Canonical Correlation Analysis Fusion

CCA fusion is used to study the linear relationship between two groups of multidimensional variables. Using two sets of signals *Y* and *X*, the goal is to find two linear projection vectors *w*_*Y*_ and *w*_*X*_ so that the two groups of linear combination signals *w*_*Y*_^*T*^ *Y* and *w*_*X*_^*T*^ *X* have the largest correlation coefficients:

(10) $$\begin{array}{c} \underset{w_{Y},w_{X}}{\max}p=\frac{E\left(w_{Y}^{T}YX^{T}w_{X}\right)}{\sqrt{E\left(w_{Y}^{T}YY^{T}w_{Y}\right)}E\left(w_{X}^{T}XX^{T}w_{X}\right)}. \end{array}$$

The reference signals were constructed at the stimulation frequency *f*_*d*_:

(11) $$\begin{array}{c} X=\left\{\begin{array}{c} \cos\left(2\pi f_{d}t\right)\\ \sin\left(2\pi f_{d}t\right)\\ \vdots \\ \cos\left(2k\pi f_{d}t\right)\\ \sin\left(2k\pi f_{d}t\right) \end{array}\right\},\;t=\frac{1}{f_{s}},\ldots ,\frac{l}{f_{s}}, \end{array}$$

where *k* is the number of harmonics, which is dependent on the number of frequency harmonics existing in SSMVEP; *f*_*s*_ is the sampling rate; and *l* represents the number of sample points. The optimization problem of equation (10) can be transformed into the eigenvalue decomposition problem, and the spatial filter coefficient *W* is the eigenvector corresponding to the largest eigenvalue (MATLAB code for CCA fusion and test data is provided in Supplementary Materials (available here)).

### 2.7. Two-Dimensional T-F Image Representation and Reconstruction of EEG Signals

The T-F analysis can transform the signal from the time domain to the T-F domain, and then, the T-F domain signal can be regarded as an image for analysis. In this study, T-F transform was performed on the time-domain EEG signal using STFT. Let *h*(*t*) be a time window function with center at *τ*, a height of 1, and a finite width. The portion of the signal *x*(*t*) observed by *h*(*t* − *τ*) is *x*(*t*)*h*(*t* − *τ*). The STFT is generated by the fast Fourier transform (FFT) of the windowed signal *x*(*t*)*h*(*t* − *τ*):

(12) $$\begin{array}{c} {\text{STFT}}_{x}\left(\tau ,f\right)=\int_{-\infty }^{+\infty }x\left(t\right)h\left(t-\tau \right)e^{-j2\pi ft}dt. \end{array}$$

Equation (12) can map the signal *x*(*t*) onto the two-dimensional T-F plane (*τ*, *f*), and *f* can be regarded as the frequency in the STFT. After fusing multiple EEG T-F images, the fused two-dimensional image signals need to be transformed into one-dimensional time-domain signals for the subsequent analysis. The STFT has an inverse transform that transforms the T-F domain signal into a time-domain signal:

(13) $$\begin{array}{c} x\left(t\right)=\int_{-\infty }^{+\infty }\int_{-\infty }^{+\infty }\text{STFT}\left(\tau ,f\right)h\left(t-\tau \right)e^{j2\pi ft}df\;d\tau . \end{array}$$

In this study, the T-F transform of the SSMVEP signal was conducted by MATLAB's *tfrstft* function. After fusing two T-F images, the T-F signal was inversely transformed into a one-dimensional time-domain signal by MATLAB's *tfristft* function.

### 2.8. T-F Image Fusion Based on Two-Dimensional Multiscale Wavelet Transform

Among the many image fusion technologies, two-dimensional multiscale wavelet transform-based image fusion methods have become a research hotspot [14–16]. Let *f*(*x*, *y*) denote a two-dimensional signal, and *x* and *y* are its abscissa and ordinate, respectively. Let *ψ*(*x*, *y*) denote a two-dimensional basic wavelet and *ψ*_*a*;*b*_1_,*b*_2__(*x*, *y*) denote the scale expansion and two-dimensional displacement of *ψ*(*x*, *y*):

(14) $$\begin{array}{c} \psi_{a;b_{1},b_{2}}\left(x,y\right)=\frac{1}{a}\psi \left(\frac{x-b_{1}}{a},\frac{y-b_{2}}{a}\right). \end{array}$$

Then, the two-dimensional continuous wavelet transform is

(15) $$\begin{array}{c} {\text{WT}}_{f}\left(a;b_{1},b_{2}\right)=\frac{1}{a}\int \int f\left(x,y\right)\psi \left(\frac{x-b_{1}}{a},\frac{y-b_{2}}{a}\right)dx\;dy. \end{array}$$

**Figure 1:**
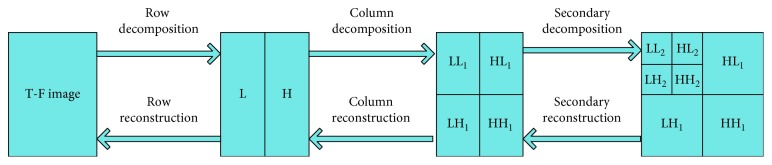
The process of two-dimensional discrete wavelet decomposition and reconstruction.

The factor 1/*a* in equation (15) is a normalization factor that has been introduced to ensure that the energy remains unchanged before and after wavelet expansion. The two-dimensional multiscale wavelet decomposition and reconstruction process is shown in Figure 1. The decomposition process can be described as follows: First, one-dimensional discrete wavelet decomposition is performed on each line of the image, which obtains the low-frequency component *L* and the high-frequency component *H* of the original image in the horizontal direction. The low-frequency component and high-frequency component are the low-frequency wavelet coefficients and high-frequency wavelet coefficients obtained after wavelet decomposition, respectively. Then, one-dimensional discrete wavelet decomposition is performed on each column of the transformed data to obtain the low-frequency components LL in the horizontal and vertical directions, the high-frequency components LH in the horizontal and vertical directions, the low-frequency components HL in the horizontal and vertical directions, and the high-frequency component HH in the vertical direction. The reconstruction process can be described as follows: Firstly, one-dimensional discrete wavelet reconstruction is performed on each column of the transform result. Then, one-dimensional discrete wavelet reconstruction is performed on each row of the transformed data to obtain the reconstructed image.

**Figure 2:**
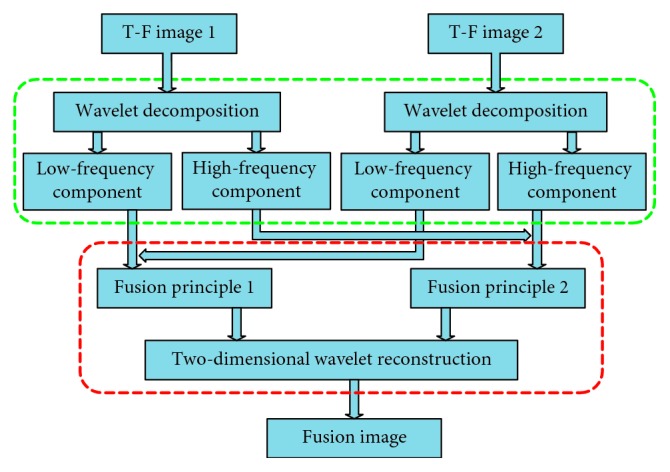
Image fusion process based on two-dimensional wavelet transform.

The T-F image obtained by the STFT is decomposed into *N* layers by the two-dimensional multiscale wavelet decomposition, as shown in Figure 1, and 3*N* + 1 frequency components are obtained. In this study, the LL_*N*_ decomposed from the highest level is defined as the low-frequency component, and the HL_*M*_, LH_*M*_, and HH_*M*_ (*M* = 1, 2,…, *N*) decomposed from each level are defined as high-frequency components. The active components of the EEG are mainly contained in the low-frequency component. The fusion process of two T-F images based on two-dimensional multiscale wavelet transform is shown in Figure 2. Firstly, the T-F images 1 and 2 are decomposed by two-dimensional wavelet decomposition, and then, the corresponding low-frequency components and high-frequency components are fused according to the corresponding fusion rules. Finally, two-dimensional wavelet reconstruction is performed to obtain the fused image by fusing low-frequency components and high-frequency components. In this study, the two-dimensional wavelet decomposition was performed using the MATLAB's *wavedec2* function and the two-dimensional wavelet reconstruction was performed using the MATLAB's *waverec2* function.

### 2.9. Image Mean Filtering

For the current pixel to be processed, a template is selected, which is composed of several pixels adjacent to the current pixel. The method of replacing the value of the original pixel with the mean of the template is called mean filtering. Defining *f*(*x*, *y*) as the pixel value at coordinates (*x*, *y*), the current pixel *g*(*x*, *y*) can be calculated by

(16) $$\begin{array}{c} g\left(x,y\right)=\frac{1}{D}\overset{D}{\underset{i=1}{\sum }}f\left(x_{i},y_{i}\right), \end{array}$$

where *D* represents the square of the template size.

### 2.10. Summary of the Proposed Method for EEG Enhancement Based on T-F Image Fusion

**Figure 3:**
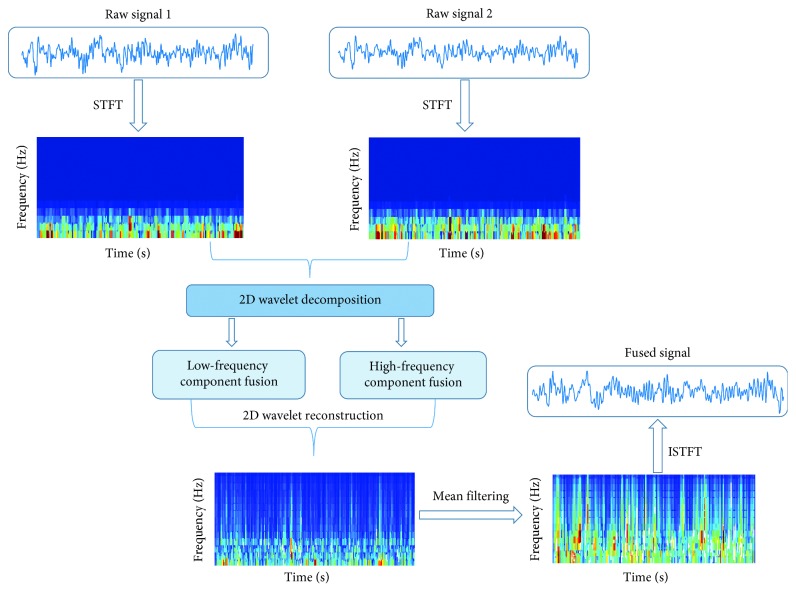
The fusion process of two-electrode signal T-F images.

Here, the T-F image fusion method of the electrode signals *x*_1_(*t*) and *x*_2_(*t*) is introduced, and the fusion process is shown in Figure 3.

1. STFT is performed on the two electrode signals *x*_1_(*t*) and *x*_2_(*t*) to obtain T-F images 1 and 2.
2. The low-frequency components LL_*N*,1_ and LL_*N*,2_ and the high-frequency components (HL_*M*,1_, LH_*M*,1_, and HH_*M*,1_) and (HL_*M*,2_, LH_*M*,2_, and HH_*M*,2_) (*M* = 1, 2, ..., *N*) of two T-F images 1 and 2 are obtained by *N*-layer two-dimensional multiscale wavelet decomposition.
3. The low-frequency components LL_*N*,1_ and LL_*N*,2_ and the high-frequency components (HL_*M*,1_, LH_*M*,1_, and HH_*M*,1_) and (HL_*M*,2_, LH_*M*,2_, and HH_*M*,2_) (*M* = 1, 2, ..., *N*) of each decomposition layer are fused according to the following fusion rules:
  (a) The low-frequency components of the T-F images 1 and 2 are subtracted to obtain the low-frequency components of the fused image *F*:

(17) $$\begin{array}{c} {\text{LL}}_{N,F}={\text{LL}}_{N,1}-{\text{LL}}_{N,2}. \end{array}$$

  The low-frequency components represent the active components of the SSMVEP, and the subtraction of the low-frequency components can remove the common noise in the electrode signals.
  (b) The magnitudes of the high-frequency components (HL_*M*,1_, LH_*M*,1_, and HH_*M*,1_) and (HL_*M*,2_, LH_*M*,2_, and HH_*M*,2_) (*M* = 1, 2, ..., *N*) of each decomposition layer are obtained for the T-F images 1 and 2. The high-frequency component with a large value is used as the high-frequency component of the fused image *F*:

(18) $$\begin{array}{c} \left\{\begin{array}{c} {{\text{HL}}^{i}}_{M,F}=\left\{\begin{array}{c} {{\text{HL}}^{i}}_{M,1},\text{real}\left({{\text{HL}}^{i}}_{M,1}\right)>\text{ real}\left({{\text{HL}}^{i}}_{M,2}\right),\\ {{\text{HL}}^{i}}_{M,2},\text{ real }\left({{\text{HL}}^{i}}_{M,1}\right)<\text{ real }\left({{\text{HL}}^{i}}_{M,2}\right), \end{array}\right.\\ {{\text{LH}}^{i}}_{M,F}=\left\{\begin{array}{c} {{\text{LH}}^{i}}_{M,1},\text{ real }\left({{\text{LH}}^{i}}_{M,1}\right)>\text{ real}\left({{\text{LH}}^{i}}_{M,2}\right),\\ {{\text{LH}}^{i}}_{M,2},\text{ real }\left({{\text{LH}}^{i}}_{M,1}\right)<\text{ real}\left({{\text{LH}}^{i}}_{M,2}\right), \end{array}\right.\\ {{\text{HH}}^{i}}_{M,F}=\left\{\begin{array}{c} {{\text{HH}}^{i}}_{M,1},\text{real }\left({{\text{HH}}^{i}}_{M,1}\right)>\text{ real}\left({{\text{HH}}^{i}}_{M,2}\right),\\ {{\text{HH}}^{i}}_{M,2},\text{real}\left({{\text{HH}}^{i}}_{M,1}\right)<\text{ real}\left({{\text{HH}}^{i}}_{M,2}\right), \end{array}\right. \end{array}\right.\;M=1,2,\ldots ,N. \end{array}$$

  The two-dimensional signal after the STFT is a complex signal; the two-dimensional signal after two-dimensional wavelet decomposition is still a complex signal. The value used for the comparison is the real part of the complex signal. Here, *i* represents the number of frequency components of the *M* decomposition layer (i.e., the number of wavelet coefficients).
(4) According to both low-frequency components LL_*N*,*F*_ and high-frequency components (HL_*M*,*F*_, LH_*M*,*F*_, and HH_*M*,*F*_) (*M* = 1, 2, ..., *N*) of the fused image *F*, the fused image *F* is generated by two-dimensional wavelet reconstruction.
(5) Mean filtering is performed on the fused image *F*.
(6) An ISTFT is performed on the mean filtered image to generate a fused time-domain signal (MATLAB code for T-F image fusion and test data is provided in Supplementary Materials (available here)).

## 3. Results

### 3.1. Parameter Selection

#### 3.1.1. STFT Parameters

In this study, the EEG time-domain signals were subjected to STFT and ISTFT, using the MATLAB library functions *tfrstft* and *tfristft*. The parameters that need to be set here are the number of frequency bins and the frequency smoothing window. If the number of frequency bins is too large, the calculation speed of the entire process will be low. In this study, the value of this parameter can be set to [32 54 76 98 120]. Since the Fourier transform of the Gaussian function is also a Gaussian function, the window function of the optimal time localization of the STFT is a Gaussian function. The EEG is a nonstationary signal [17] and requires a high time resolution of the window function; that is, the window length should not be too long. In this paper, a Gaussian window was selected, and the value of the window length can be set to [37 47 57 67 77 87 97].

#### 3.1.2. Two-Dimensional Multiscale Wavelet Transform Parameters

In this study, the MATLAB library functions *wavedec2* and *waverec2* were used to perform two-dimensional multiscale wavelet decomposition and reconstruction on T-F images. Here, the number of layers of wavelet decomposition and the wavelet basis functions need to be set. The sampling frequency of the EEG acquisition device used in this paper was 1200 Hz, and the stimulation frequency of the SSMVEP was 7–10.8 Hz. Considering that the SSMVEP may contain the second harmonic of the stimulation frequency, this study sets the number of layers of wavelet decomposition to 4. The db_*N*_ wavelet has good regularity and was chosen as the wavelet basis function. The vanishing moment *N* of the wavelet function can be set to [2 3 5 7 10 13].

#### 3.1.3. Mean Filtering Parameters

Here, the template size of the mean filtering needs to be set. In this study, the value of the template size can be set to [2 4 6 8 10 12 14 16 18 20 22 24 26].

#### 3.1.4. Low-Frequency Component of the Fused Image *F*

The values of LL_*N*,*F*_ taking LL_*N*,1_ *−*LL_*N*,2_ or LL_*N*,2_ *−*LL_*N*,1_ will affect the experimental results, and the values of LL_*N*,*F*_ need to be determined to optimize the final fusion effect (see details in Section 2.10 (3)).

#### 3.1.5. Parameter Selection

The T-F image fusion method proposed in this study requires the setting of the following five parameters: the number of frequency bins, frequency smoothing window, vanishing moments of db_*N*_ wavelet functions, mean filter template size, and the value of LL_*N*,*F*_. In this study, the grid search method was used to traverse the combination of all parameters to find the best combination of parameters that can enhance the SSMVEP active components. In this study, six rounds of experiments were conducted. The first three rounds of experimental data were used to select the optimal combination of parameters for T-F image fusion. According to the selected combination of parameters, the last three rounds of experimental data were used to compare the enhancement effect of SSMVEP active components by the method of minimum energy fusion, maximum contrast fusion, CCA fusion, and T-F image fusion. The disadvantage of grid search is that the amount of calculation is large; however, considering the difficulty of superparameter selection, grid search is still a more reliable method.

### 3.2. Electrode Signal Selection for T-F Image Fusion

The T-F image fusion method analyzes two electrode signals. Thus, two suitable electrodes need to be selected from multiple electrodes. The bipolar fusion signal is obtained by subtracting the two original EEG signals. In equation (17), we subtracted the wavelet low-frequency components of the two T-F images to obtain the wavelet low-frequency components of the fused image. The subtraction of the low-frequency components removes the common noise in the electrode signals, which is similar to the principle of bipolar fusion. Therefore, the two electrode signals with the best bipolar fusion effect were used in the T-F image fusion. For SSVEP or SSMVEP stimulation, the Oz electrode usually has the strongest response [18] and is most commonly used to analyze SSVEP or SSMVEP [19]. The Oz electrode was used as one of the analysis electrodes for bipolar fusion. In this study, the Oz electrode was fused with the other five electrode signals so that the best combination of electrodes can be selected. When bipolar fusion is used, the Oz electrode as a reducing electrode or a reduced electrode will not affect the experimental results. For example, Oz-POz and POz-Oz have the same fusion effect. In this study, the Oz electrode was used as the reduced electrode. Twenty trials per subject were used for electrode selection. The Welch power spectrum analysis [20] was performed on the obtained signals of bipolar fusion for each subject. The Gaussian window was used, and the length of the window was 5 s. The overlapping was 256, and the number of discrete Fourier transform (DFT) points was 6000 for the Welch periodogram. 20 focused targets correspond to 20 stimuli frequencies, and the frequencies corresponding to the highest power spectrum amplitudes at the 20 frequencies were determined as the focused target frequency. The high recognition accuracy indicates that the amplitude of the power spectrum at the stimulation frequency is prominent, and it indicates the effectiveness of the fusion method for the enhancement of the SSMVEP active component.  Table 1 shows the recognition accuracy of all 10 subjects under the bipolar fusion. The Oz electrode was fused with different electrodes and obtained different fusion effects. Moreover, due to the differences among individuals, the electrode combination with the highest recognition accuracy per subject was also different. The asterisk (^*∗*^) marks the bipolar fusion electrode group with the highest recognition accuracy of each subject, and the corresponding electrode combination was used in T-F image fusion.

### 3.3. Comparison of Enhancement Effects of Minimum Energy Fusion, Maximum Contrast Fusion, CCA Fusion, and T-F Image Fusion on SSMVEP Active Components

**Figure 4:**
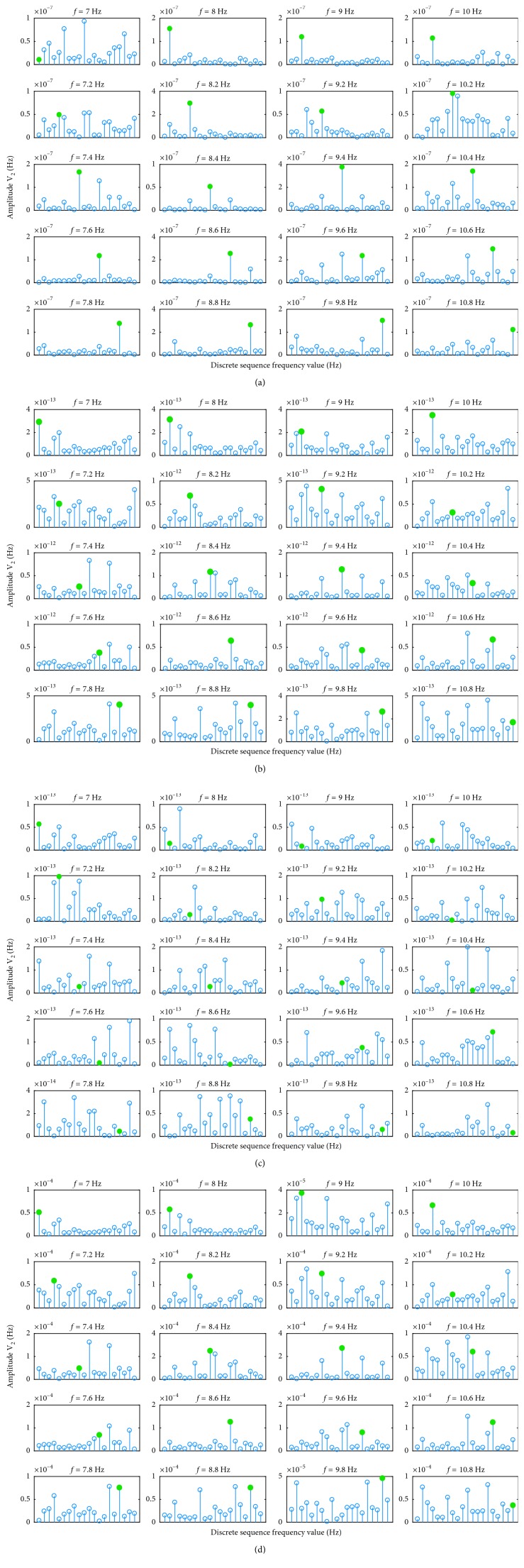
The power spectrum results of 20 targets under (a) T-F image fusion, (b) CCA fusion, (c) minimum energy fusion, and (d) maximum contrast fusion (the discrete sequence frequency values from left to right are {7 8 9 10 7.2 8.2 9.2 10.2 7.4 8.4 9.4 10.4 7.6 8.6 9.6 10.6 7.8 8.8 9.8 10.8} Hz, respectively).

T-F image fusion used two channel signals, and CCA fusion, maximum contrast fusion, and minimum energy fusion used all six channel signals. In this study, the accuracy was used as the evaluation standard of the fusion effect. The high online accuracy indicates that the amplitude at stimulus frequency is prominent, which shows the effectiveness of the fusion method. For T-F image fusion, the Welch power spectrum analysis (the parameters are the same as in Section 3.2) was performed on the obtained signal after the T-F image fusion, and the frequency with the highest amplitude at twenty stimulus frequencies was identified as the focused target frequency. The minimum energy fusion, maximum contrast fusion, and CCA fusion require prior knowledge of the frequency of the signal to be fused. However, the frequency of the signal to be fused cannot be determined because the user's focused target cannot be determined beforehand. For the current tested signal, the minimum energy fusion, maximum contrast fusion, and CCA fusion need to first perform the fusion analysis at all stimulus frequencies, and then, the frequency with the highest amplitude was identified as the focused target frequency. Take CCA fusion as an example, and assume that the current tested signal is *Y*. First, 20 template signals are set according to equation (11), and then, 20 spatial filter coefficients are obtained according to equation (10). The tested signal is fused with 20 spatial filter coefficients, respectively. The obtained 20 sets of vectors are separately analyzed by the Welch power spectrum (the parameters are the same as in Section 3.2) to obtain the amplitude at the corresponding frequency. Finally, the frequency with the highest amplitude is identified as the focused target frequency. The first three rounds of experimental data were used to select the parameters of the T-F image fusion (see details in Section 3.1), and the last three rounds of the experimental data were used to test the online fusion effects under the four methods. Figures 4(a)–4(d) show the analysis results plotted using the data of subject 4 in the fourth round of the experiment.  Figure 4(a) shows the power spectrum results of the 20 targets under T-F image fusion, Figure 4(b) shows the power spectrum results of 20 targets under CCA fusion, Figure 4(c) shows the power spectrum results of 20 targets under minimum energy fusion, and Figure 4(d) shows the power spectrum results of 20 targets under maximum contrast fusion. In the stalk plot, the discrete sequence frequency values from left to right are {7 8 9 10 7.2 8.2 9.2 10.2 7.4 8.4 9.4 10.4 7.6 8.6 9.6 10.6 7.8 8.8 9.8 10.8}, respectively, where *f* represents the stimulation frequency of the corresponding target and the green dot marks the amplitude of the corresponding target stimulus frequency. The red flag *f* represents the target was misidentified, and the blue flag *f* represents the target was correctly identified. Figures 4(a)–4(d) show that the T-F image fusion method has the best online performance and the minimum energy fusion method has the worst online performance. Maximum contrast fusion and CCA fusion have similar fusion effects.

**Figure 5:**
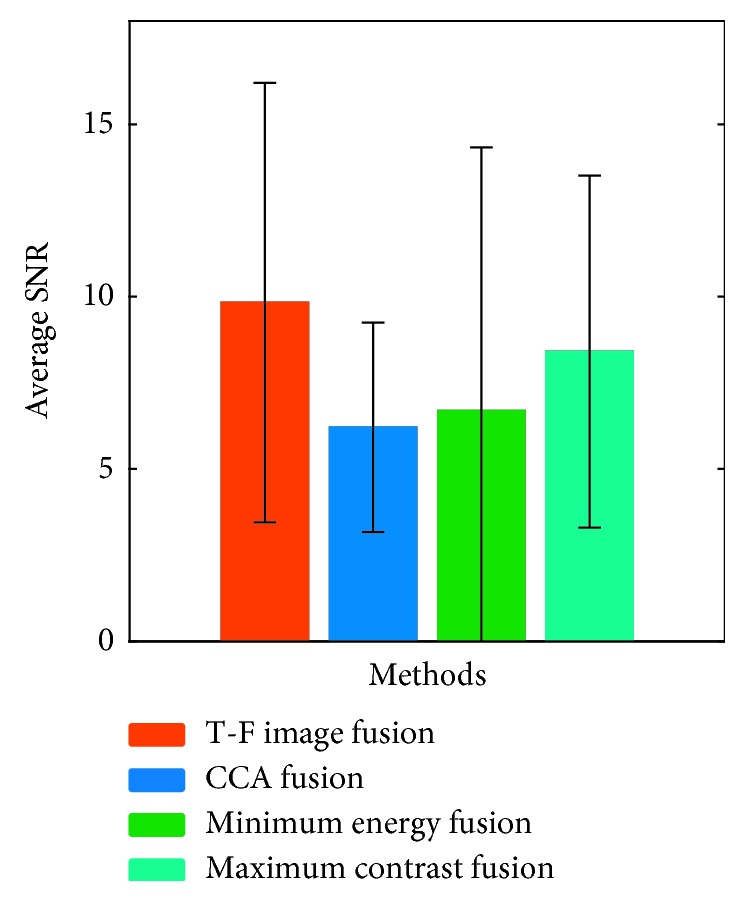
The average SNR of ten subjects at all stimulus frequencies under four methods.

In online analysis, the power spectrum amplitudes of the test signal at all stimulus frequencies were calculated, and then, the frequency corresponding to the maximum amplitude was identified as the focused target frequency. Error recognition occurs when the amplitude at the focused target frequency is lower than the amplitudes at the nonfocused target frequencies. Therefore, the power spectrum amplitude at the focused target frequency can be regarded as the active component of the signal, and the power spectrum amplitudes at the remaining nonfocused target frequencies can be regarded as noise. The SNR in this study was defined as the ratio of the power spectrum amplitude at the focused target frequency to the mean of the power spectrum amplitudes at the remaining nonfocused target frequencies. The SNRs of ten subjects at all stimulus frequencies were superimposed and averaged, and the experimental results are shown in Figure 5. It can be seen from Figure 5 that the T-F image fusion method obtained the highest SNR, which indicates that the T-F image fusion method proposed in this study can effectively enhance the SSMVEP SNR.

**Figure 6:**
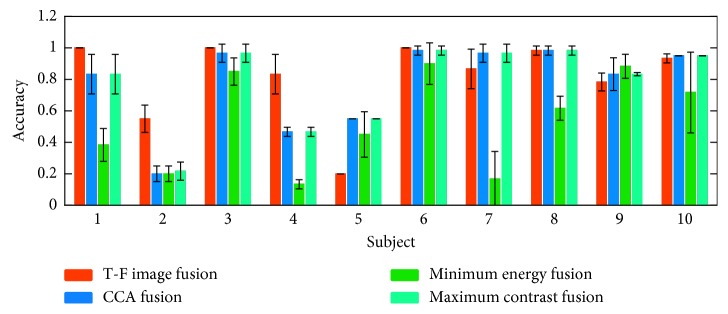
The accuracy of each subject under four fusion methods.

**Figure 7:**
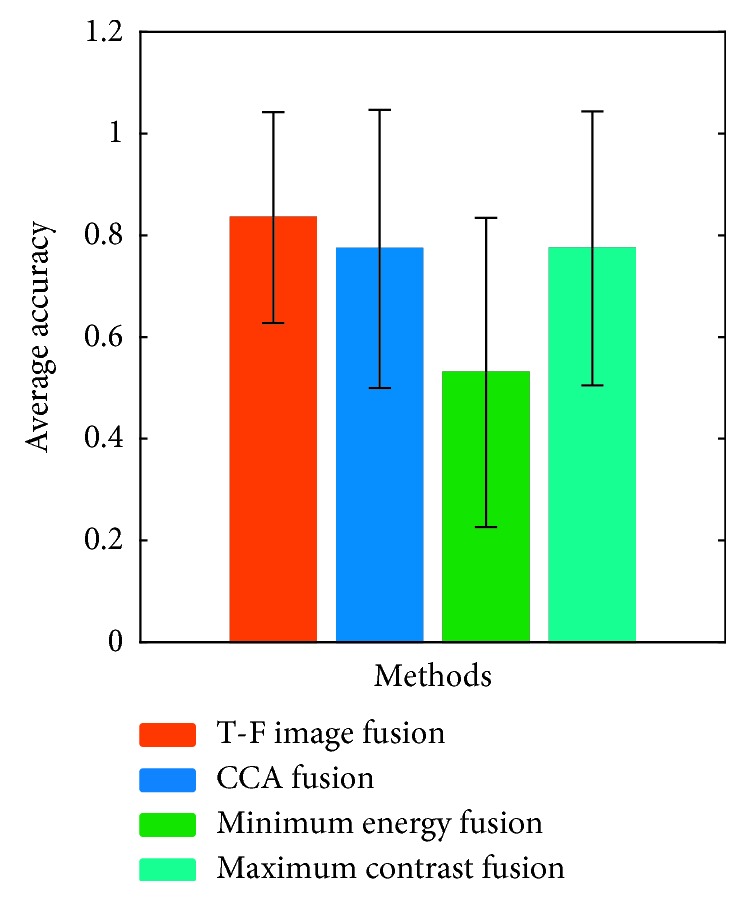
The average accuracy of ten subjects under four fusion methods.

The online accuracies of each subject in the three rounds of experiments were superimposed and averaged. The experimental results are shown in Figure 6. It can be seen that most subjects obtained highest online accuracy under the T-F image fusion and some subjects obtained highest online accuracy under CCA fusion and maximum contrast fusion. Only subject 9 obtained the highest online accuracy under the minimum energy fusion. The online accuracies of all the subjects were superimposed and averaged. The experimental results are shown in Figure 7. The experimental results show that the online performance of the T-F image fusion method is better. The online accuracy of the T-F image fusion method is 6.17%, 6.06%, and 30.50% higher than that of the CCA fusion, maximum contrast fusion, and the minimum energy fusion, respectively. The paired *t*-test shows significant differences between the accuracies of T-F image fusion and minimum energy fusion (*p*=0.0174). Online accuracy analysis results show that the proposed method is better than the traditional time-domain fusion method.

## 4. Discussion

The SSMVEP collected from the scalp contains a lot of noise and requires an effective signal processing method to enhance the active components of SSMVEP. Spatial filtering methods utilize EEG information of multiple channels and exert positive significance to enhance the active components of the SSMVEP. At present, the commonly used spatial filtering methods include average fusion, native fusion, bipolar fusion, Laplacian fusion, CAR fusion, CCA fusion, minimum energy fusion, and maximum contrast fusion. The first five spatial filtering methods can only process the preselected electrode signals. Minimum energy fusion, maximum contrast fusion, and CCA fusion improve the above problem, and they can be used to fuse any number of electrode signals with better fusion effects. Minimum energy fusion, maximum contrast fusion, and CCA fusion fuse SSMVEP in the time domain. This study first put forward the idea of fusing EEG in the T-F domain and proposed a T-F image fusion method. We compared the enhancement effects of minimum energy fusion, maximum contrast fusion, CCA fusion, and T-F image fusion on SSMVEP. It is verified by the test data that the proposed method is better than the traditional time-domain fusion method.

T-F analysis is a good choice for the transformation of the EEG data from one dimension to two dimension. EEG transformed into the T-F domain can be used as an image for analysis. The STFT has an inverse transform, which can inverse transform the fused T-F image into a time-domain signal. Therefore, the STFT was used to transform the EEG from the time domain to the T-F domain. The two-dimensional wavelet transform can fuse two images into one to achieve the purpose of multichannel EEG fusion. After two-dimensional wavelet decomposition, the low-frequency components of the two subimages were subtracted to obtain the low-frequency components of the fused image, and the maximum value of the high-frequency components of the two subimages was taken as the high-frequency component of the fused image. The low-frequency components after wavelet decomposition represent the active components of the SSMVEP. The subtraction of the low-frequency components can remove the common noise in the electrode signals. This is similar to bipolar fusion, which subtracts two electrode signals in the time domain. We also compared the enhancement effects of bipolar fusion and the proposed method on SSMVEP active components. The electrodes used for bipolar fusion are the same as those used in T-F image fusion. The results show that the proposed method is better than the bipolar fusion (the online accuracy of bipolar fusion is 71.52%). In this study, the fused image was filtered by mean filter to further achieve low-pass filtering. We also tried to remove the mean filtering step after completing the T-F image fusion. The results show that removing the mean filtering step reduced the fusion effect. In this study, the mean filtering step was performed after T-F image fusion. We tested the fusion effect when the mean filtering step was performed before T-F image fusion (performing mean filtering step on the subimage after STFT). For some subjects, it was better to perform the mean filtering step after T-F image fusion. Therefore, we recommend that researchers follow the analysis steps in this study.

**Figure 8:**
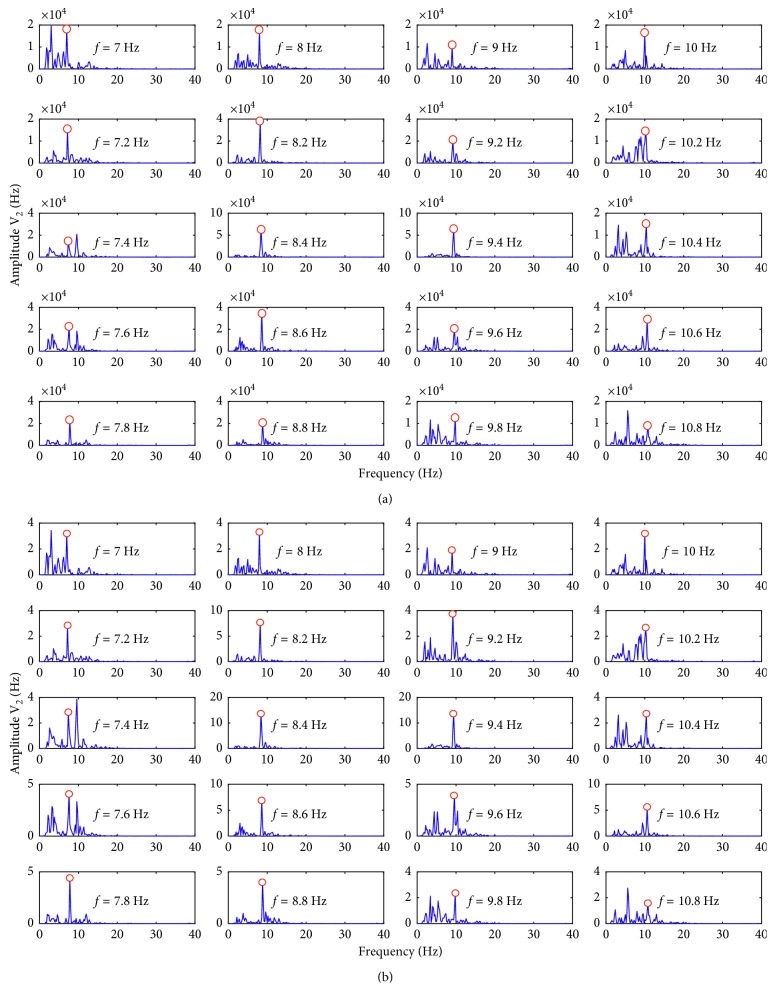
The Welch power spectrum plotted using the (a) CCA fusion spatial filter coefficients and (b) maximum contrast fusion spatial filter coefficients.

The wavelet low-frequency coefficients (see details in equation (17)) of the fused image have an important influence on the fusion effect. If we perform T-F image fusion on six electrode signals at the same time, we will get six sets of wavelet low-frequency coefficients. Here, each set of low-frequency coefficient is a two-dimensional signal. Referring to equation (1), we can assign a coefficient to each two-dimensional signal and obtain the fused two-dimensional signal (i.e., the wavelet low-frequency coefficients of the fused image) after linear summation. This study explored the feasibility of applying the spatial filter coefficients of CCA fusion and maximum contrast fusion to T-F image fusion when fusing six electrode signals by using the T-F image fusion method at the same time. Since the fusion effect of minimum energy fusion was poor, the spatial filter coefficients of the minimum energy fusion were not used here. The spatial filter coefficients of the maximum contrast fusion and CCA fusion at the frequency *f* were obtained by equations (9) and (10) and are set to *V*_max_ and *V*_cca_, respectively. *V*_max_ and *V*_cca_ are two vectors with dimensions of 6 × 1, where *V*_max_(1, 1) and *V*_cca_(1, 1) represent the first elements of the vectors *V*_max_ and *V*_cca_. Since the T-F image fusion method was performed on the six electrode signals, equation (17) is transformed into equation (19), where *V* corresponds to the spatial filter coefficient *V*_max_ or *V*_cca_. The rest of the analysis process is the same as that in Section 2.10. The same analysis steps were performed for all 20 targets (7–10.8 Hz). The Welch spectrum analysis (the parameters are the same as in Section 3.2) was performed on the obtained signal after T-F image fusion. Figures 8(a) and 8(b) show the Welch power spectra plotted using the data of subject 4 in the fourth round of the experiment.  Figure 8(a) shows the power spectrum plotted using spatial filter coefficients of CCA fusion and Figure 8(b) shows the Welch power spectrum plotted using spatial filter coefficients of maximum contrast fusion, where *f* represents the stimulus frequency and the red circle indicates the amplitude at the stimulus frequency. Figures 8(a) and 8(b) show that the amplitudes at the stimulation frequencies are prominent. The spatial filter coefficients of CCA fusion and maximum contrast fusion are effective for T-F image fusion. Thus, the T-F image fusion method proposed in this study focuses on the fusion of wavelet low-frequency coefficients of multiple images (by assigning a coefficient to each two-dimensional signal and obtaining the fused two-dimensional signal after linear summation). The premise of the above test results is that we know the frequency of the signal to be fused. If the online test method is used (see details in Section 3.3), the same online fusion results as CCA fusion and maximum contrast fusion were obtained. T-F image fusion used only two electrode signals, and the better online fusion effect than that of the CCA fusion and the maximum contrast fusion was obtained, which shows the effectiveness of the proposed method. Next, we will explore the fusion method of multiple T-F images (more than two images), that is, to find suitable spatial filter coefficients for the wavelet low-frequency components of multiple T-F images:

(19) $$\begin{array}{c} {\text{LL}}_{N,F}={\text{LL}}_{N,1}*V\left(1,1\right)+{\text{LL}}_{N,2}*V\left(2,1\right)+{\text{LL}}_{N,3}*V\left(3,1\right)+{\text{LL}}_{N,4}*V\left(4,1\right)+{\text{LL}}_{N,5}*V\left(5,1\right)+LL_{N,6}*V\left(6,1\right). \end{array}$$

The parameters affect the fusion effect of the proposed method. We listed the parameters that need to be selected and the possible values of the parameters in Section 3.1. In this study, the grid search method was used to traverse the combination of all parameters, and the best combination of parameters was found. In the experiment, we found that the number of frequency bins and the Gaussian window length can be fixed to 54 and 57. We recommend that the researchers determine the parameters according to the parameter selection principles and ranges given in Section 3.1. In this study, the SSMVEP active component was enhanced by fusing multichannel signals into a single-channel signal, and then, the focused target frequency was identified by performing spectrum analysis on the fused signals. This is beneficial for SSMVEP studies based on spectrum analysis. For example, Reference [21] proposed a frequency and phase mixed coding method in the SSVEP-based brain-computer interface (BCI), which increases the number of BCI coding targets by making one frequency correspond to multiple different phases. In the study, the FFT analysis of the test signal is required to find the possible focused target frequency and then calculate the phase value at that frequency. The proposed method in this study has a positive significance for accurately finding the focused target frequency in the spectrum. Moreover, some EEG feature extraction algorithms for the BCI also require spectrum analysis of the test signals [22, 23]. Therefore, the method proposed in this study has a potential application value.

## 5. Conclusion

To explore whether T-F domain analysis can achieve better fusion effects than time-domain analysis, this study proposed an SSMVEP enhancement method based on T-F image fusion. The parameters of the T-F image fusion algorithm were determined by the grid search method, and the electrode signals used for T-F image fusion were selected by bipolar fusion. The analysis results show that the key of the T-F image fusion algorithm is the fusion of the wavelet low-frequency components. This study compared the enhancement effects of minimum energy fusion, maximum contrast fusion, CCA fusion, and T-F image fusion on SSMVEP. The experimental results show that the online performance of the T-F image fusion method is better than that of the traditional spatial filtering methods, which indicates that the proposed method is feasible to fuse SSMVEP in the T-F domain.

## Figures and Tables

**Table 1:** Accuracies of 10 subjects under bipolar fusion.

Subjects	Bipolar fusion				
	Oz-PO7	Oz-PO8	Oz-PO3	Oz-POz	Oz-PO4
S1	0.2	0.7	0.45	0.15	0.95^*∗*^
S2	0.25	0	0.3^*∗*^	0.1	0.2
S3	0.95^*∗*^	0.85	0.85	0.4	0.8
S4	0.8^*∗*^	0.1	0.7	0.35	0.35
S5	0.2^*∗*^	0.15	0.15	0.15	0.1
S6	0.95^*∗*^	0.3	0.95^*∗*^	0.85	0.7
S7	0.2	0.15	0.25	0.6^*∗*^	0.55
S8	0.1	0.05	0.25	0.3	0.9^*∗*^
S9	0.3	0.15	0	0.35^*∗*^	0.2
S10	0.35	0.35	0.5	0.8^*∗*^	0.4

^*∗*^The bipolar fusion electrode group with the highest recognition accuracy of each subject.

## Data Availability

The analytical data used to support the findings of this study are included within the article, and the raw data are available from the corresponding author upon request.

## Supplementary Materials

Supplementary Materials MATLAB codes for T-F image fusion, minimum energy fusion, maximum contrast fusion, and CCA fusion.
